# Influencing Factors on Radiotherapy Outcome in Stage I-II Glottic Larynx Cancer—A Multicenter Study

**DOI:** 10.3389/fonc.2019.00932

**Published:** 2019-09-20

**Authors:** Olgun Elicin, Ekin Ermiş, Christoph Oehler, Daniel M. Aebersold, Francesca Caparrotti, Frank Zimmermann, Gabriela Studer, Guido Henke, Lukas Adam, Lukas Anschuetz, Mahmut Ozsahin, Matthias Guckenberger, Mohamed Shelan, Nuri Kaydıhan, Oliver Riesterer, Robin J. D. Prestwich, Thierry Spielmann, Roland Giger, Mehmet Şen

**Affiliations:** ^1^Department of Radiation Oncology, Inselspital, Bern University Hospital, University of Bern, Bern, Switzerland; ^2^Department of Clinical Oncology, Leeds Cancer Center, St. James's Institute of Oncology, Leeds, United Kingdom; ^3^Department of Radiation Oncology, Cantonal Hospital of Graubunden, Chur, Switzerland; ^4^Department of Radiation Oncology, University Hospital of Geneva, Geneva, Switzerland; ^5^Department of Radiation Oncology, University Hospital Basel, Basel, Switzerland; ^6^Department of Radiation Oncology, Cantonal Hospital of Lucerne, Lucerne, Switzerland; ^7^Department of Radiation Oncology, University Hospital of Zurich, Zurich, Switzerland; ^8^Department of Radiation Oncology, Cantonal Hospital of St. Gallen, St. Gallen, Switzerland; ^9^Department of Otorhinolaryngology, Head and Neck Surgery, Inselspital, Bern University Hospital, University of Bern, Bern, Switzerland; ^10^Department of Radiation Oncology, University Hospital of Lausanne, Lausanne, Switzerland; ^11^Department of Radiation Oncology, Cerrahpaşa Faculty of Medicine, Istanbul University, Istanbul, Turkey

**Keywords:** larynx cancer, radiotherapy, squamous cell carcinoma, sex, head and neck cancer

## Abstract

**Background and Purpose:** Larynx cancer represents one of the most frequently diagnosed head and neck malignancies, which is most often confined to the glottic area. The aim of this study was to report the oncological outcome and identify prognostic factors in early-stage glottic squamous cell carcinoma treated with radiotherapy.

**Material and Methods:** Patients (*n* = 761) diagnosed and treated in 10 centers between 1990 and 2015 were retrospectively analyzed. Probabilities of loco-regional control (LRC) and overall survival (OS) were calculated and possible prognostic factors were analyzed using Cox proportional hazards models.

**Results:** The median follow-up was 63 months (range: 2–243). Three hundred and sixty-four, 148 and 249 patients had cT1a, cT1b, and cT2 stage I-II disease, respectively. Five and 10-years LRC/OS rates in the whole cohort were 83/82% and 80/68%, respectively. Three patients developed distant recurrences. In univariate analysis, male sex (HR: 3.49; 95% CI: 1.47–11.37; *p* < 0.01), T2 vs. T1a (HR: 1.62; 95% CI: 1.08–2.43; *p* = 0.02) and anterior commissure involvement (ACI) (HR: 1.66; 95% CI: 1.38–2.45; *p* < 0.01) were associated with impaired LRC. In multivariate analysis, male sex (HR: 3.42; 95% CI: 1.44–11.17; *p* < 0.01) and ACI (HR: 1.51; 95% CI: 1.01–2.28; *p* = 0.047) remained poor prognostic factors. No relation of treatment technique and biologically equivalent dose (BED) to oncological outcome was identified except for higher BED_10_(L = 25; T = 1) yielding better LRC in T1a tumors (*p* = 0.04) in univariate analyses.

**Conclusion:** Our results highlight the negative impact of ACI on tumor control. A less-expected finding was the impact of sex on tumor control. Further research is needed to validate its prognostic value and investigate any related biologic or behavioral factors, which may be modified to improve oncologic outcome.

## Introduction

Laryngeal squamous cell carcinoma comprises around 25% of all head and neck cancers (1). About 50–60% of the laryngeal squamous cell carcinoma arise from the glottic region (2) and over 80% of those patients present in an early UICC (Union for International Cancer Control) stage (3). The larynx has important roles in production of phonation, coordination of swallowing and respiration. Therefore, the treatment aim of laryngeal cancer is not only achieving maximum disease control, but also preservation of organ function. In the absence of large randomized studies providing a clear evidence for the best strategy to treat early-stage glottic squamous cell carcinoma (EGSCC), many retrospective studies reported comparable control rates following radiotherapy (RT) or surgery. The 5-year loco-regional control (LRC) following RT ranges from 80 to 95% for T1 and 61–82% for the T2 cancer. Five year overall (OS) rates in stage I and II are in the range of 89–100% and 60–100%, respectively (4, 5).

The negative prognostic factors reported for EGSCC are higher stage (6), anterior commissure involvement (ACI) (7–11), anemia (12), continuation of smoking (13), and protracted treatment time (14). Amongst these, the impact of dose/fractionation has been extensively investigated. Multiple randomized studies have demonstrated a benefit of shorter treatment time, regardless of whether that goal was achieved by means of acceleration or hypofractionation (15–19). Current recommendation in the national guidelines is to treat EGSCC with fraction sizes of 2 Gy up to 66 (stage I) - 70 Gy (stage II) preferably in an accelerated schedule or with hypofractionation, with fraction sizes such as 2.25 Gy up to 63 Gy (stage I) – 65.25 Gy (stage II) and 2.75 Gy to a dose of 55 Gy (20–23). On the other hand, there are also published works with findings contradicting with the above-mentioned information (24, 25). Furthermore, there seem to be other factors such as sex, which were suggested to influence tumor control (26, 27) and survival (28, 29).

The aim of this retrospective study was to assess the oncologic outcome and its potentially influencing factors after RT of stage I and II EGSCC in a multicenter setting with a sufficient follow-up.

## Materials and Methods

Approvals of institutional and regional review boards were obtained. All subjects gave written informed consent in accordance with the Declaration of Helsinki. The charts of all patients diagnosed and treated with EGSCC in 10 university and teaching hospitals from three countries (Switzerland, Turkey and United Kingdom) between 1990 and 2015 with histologically-proven stage I and II invasive EGSCC were reviewed. All patients underwent endoscopic examination of the upper airways under general anesthesia. The diagnostic workup of the stage II tumors was completed with a magnetic resonance imaging or computed tomography of the neck, and computed tomography or X-ray of the chest based on the year and institution. Imaging workup for staging of T1 tumors was not standard in every site throughout the years the patients were diagnosed. Previously treated (i.e., surgery or radiation) cases were excluded. Surgical excisions with a failed aim of achieving clear margins (i.e., with an indication of adjuvant radiotherapy) were also excluded. Excisional biopsies with a diagnostic intent were allowed.

The centers were arbitrarily selected via personal communication. Patient and disease characteristics, such as age, sex, date of diagnosis (date of initial positive biopsy), tumor stage, presence of ACI and treatment features, such as start and end date of RT, dose/fractionation and treatment technique were collected. Regarding follow-up, information about relapse, mortality, incidence and localization of second primary cancer (SPC) were obtained. Staging was revised according to the 8th edition of UICC staging system (30). Information about RT portals and target volumes, smoking, alcohol abuse, toxicity and complications was not obtained.

RT was delivered using two-dimensional conventional or three-dimensional conformal technique in the majority of the cases, followed by an era of intensity modulated radiotherapy (IMRT). The follow-up schedules and assessment measures of toxicity were not standard among all centers.

Due to the expected heterogeneity in dose and fractionation schedules among centers, two distinct biologically equivalent dose (BED) models were generated by using the following equation (31): BED_α/β_ = D(1 + d/(α/β)) – (OTT – L) x T [*D: total dose; d: dose per fraction; OTT: overall treatment time; L: time lag; T: time factor*]. Based on L and T published in the literature (31–33), two biological scenarios were simulated: BED_10_(L = 25; T = 1) and BED_10_(L = 28; T = 0.6). BED_10_(L = 25; T = 1) corresponds to a rather aggressive tumor biology, by which the accelerated repopulation starts after day 25, and afterwards the daily loss of dose regarding tumor control probability is 1 Gy. On the other hand, BED_10_(L = 28; T = 0.6) refers to a relatively less aggressive tumor biology, by which the accelerated repopulation starts after day 28, and the daily loss of dose is 0.6 Gy.

All time-to-event intervals were calculated based on the date of initial positive biopsy. The follow-up time was not censored at a predefined time point. Kaplan-Meier curves and log-rank test were used to depict and compare the variables regarding time-to event endpoints, respectively. Univariate Cox's proportional hazards regression was used to evaluate possible prognostic factors including age, sex, T stage, ACI, BED_10_ and treatment modality for LRC. Variables yielding two-sided *p* < 0.1 were used to build multivariate models. Backwards elimination was used to identify potential independent factors. Statistical analyses were performed with JMP (version 14.0 - SAS Institute GmbH, Germany). The anonymized version of the data will be provided upon reasonable personal request.

## Results

Seven hundred sixty-one patients from three countries were diagnosed and treated at 10 institutions in a timeframe of 25 years. The median follow-up was 63 months (range: 2–243). Table 1 summarizes the patient, disease and treatment characteristics (details of treatment characteristics provided in the Supplementary Materials 1, 2). The reasons for discontinuing with follow-up varied from patient preference to outsourcing the follow-up controls to an external ear, nose and throat specialist in some centers.

**Table 1:** Patient, disease, and treatment characteristics.

**Characteristics**	**Whole cohort** **(*n* = 761)**	**Stage T1a** **(*n* = 364/48%)**	**Stage T1b** **(*n* = 148/19%)**	**Stage T2** **(*n* = 249/33%)**
Median age in years (range)	65 (33-97)	64 (35-97)	68 (38-96)	65 (33-95)
**Gender**				
Male	685 (90%)	331 (91%)	130 (88%)	224 (90%)
Female	76 (10%)	33 (9%)	18 (12%)	25 (10%)
**Anterior commissure involvement**				
Yes	414 (54%)	124 (34%)	119 (80%)	171 (69%)
No	347 (46%)	240 (66%)	29 (20%)	78 (31%)
**Radiotherapy technique**				
2D-/3D-RT	604 (79%)	301 (83%)	117 (79%)	186 (75%)
IMRT	157 (21%)	63 (17%)	31 (21%)	63 (25%)
Median number of fractions (range)	34 (16-68)	34 (16-62)	34 (16-63)	35 (20-68)
Median fraction size in Gy (range)	2 (1.18–3.14)	2 (1.2–3.14)	2 (1.18–3.14)	2 (1.18–2.75)
Median total dose in Gy (range)	68 (50–81.6)	68 (50.24–78.12)	68 (50–78.3)	70 (55–81.6)
Median treatment time in days (range)	45 (21-80)	46 (21-80)	46 (22-60)	43 (25-70)
BED_10_(L = 25; T = 1) median in Gy (range)	61.4 (29-77)	60.1 (29-77)	61 (49-77)	64.6 (36.6–74.6)
BED_10_(L = 28; T = 0.6) median in Gy (range)	70.8 (52.8–83.4)	70.6 (52.8–83.4)	70.8 (64.8–81.6)	71.9 (54.2–79.8)

*2D-RT, two-dimensional radiotherapy; 3D-RT, three-dimensional conformal radiotherapy; BED, biologically equivalent dose; IMRT, intensity-modulated radiotherapy; RT, radiotherapy*.

**Figure 1:**
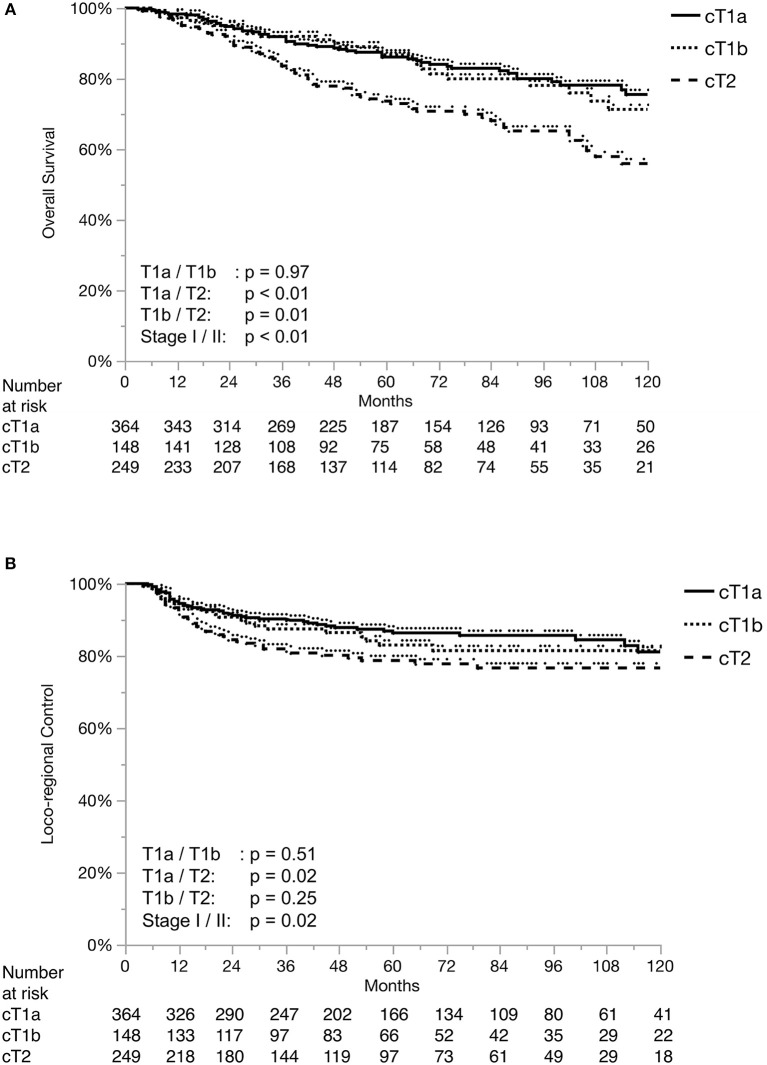
Overall Survival **(A)** and Loco-regional control **(B)** separated by T stage.

**Figure 2:**
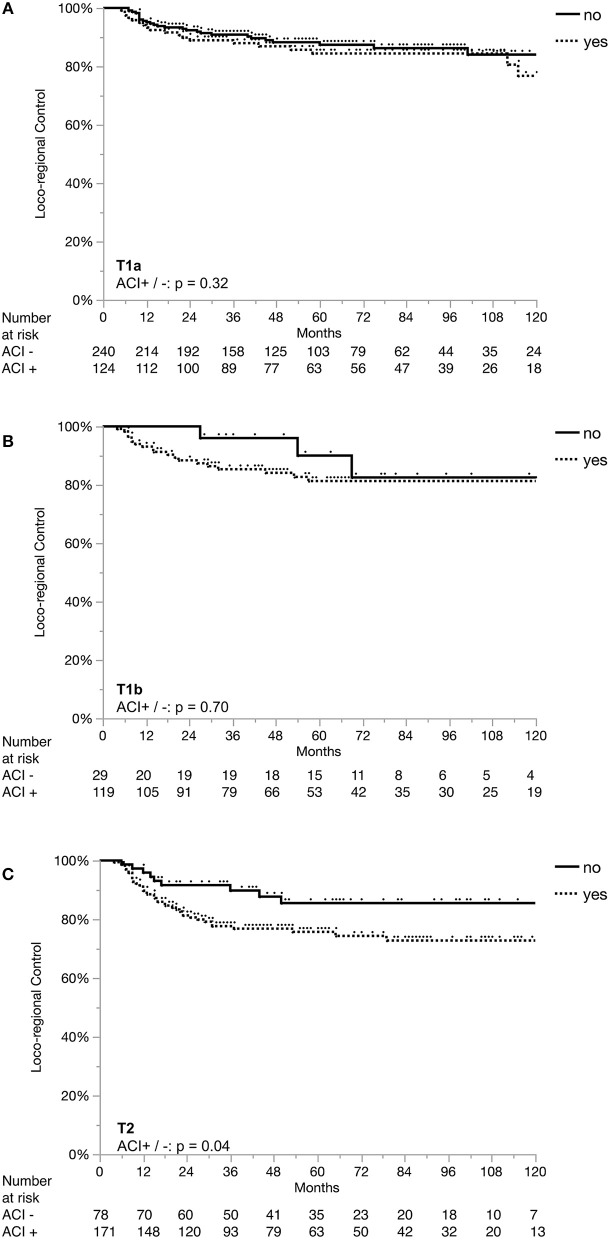
Loco-regional control separated by anterior commissure involvement in each stage. ACI, anterior commissure involvement; Loco-regional control in Stage T1a **(A)**, in Stage T1b **(B)**, and in Stage T2 **(C)**.

Loco-regional control (LRC) at 2, 5 and 10 years were 89, 83, and 80%, respectively. Overall survival (OS) at 2, 5, and 10 years were 93, 82, and 68%, respectively. Figure 1 shows the Kaplan-Meier curves for OS and LRC in patients with T1a, T1b, and T2 stage tumors. Figure 2 demonstrates the separation of Kaplan-Meier curves for LRC based on ACI for each T stage.

**Figure 3:**
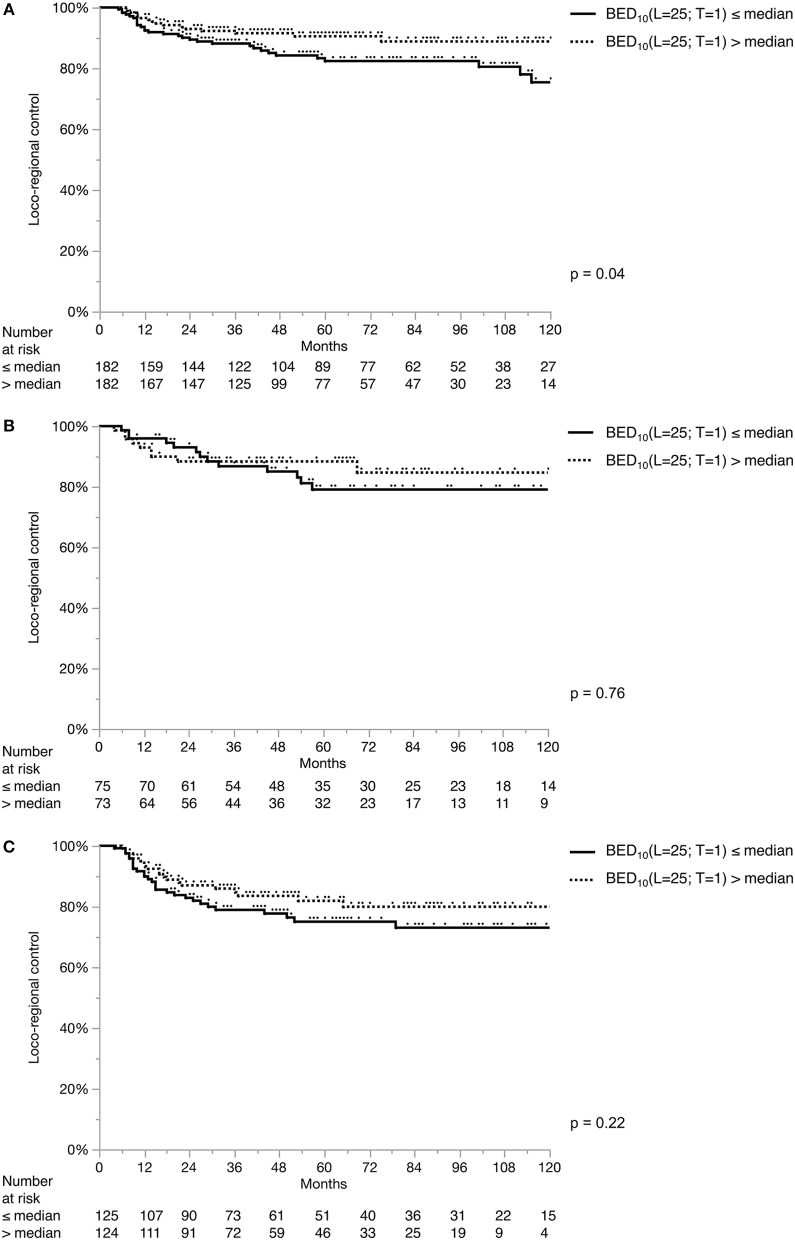
Loco-regional control separated by median BED_10_(L = 25; T = 1). Loco-regional control in T1a **(A)**, T1b **(B)**, and T2 **(C)** tumors. Each stage subgroup is dichotomized with its own median value: stage T1a: 60.1 Gy; stage T1b: 61 Gy; stage T2: 64.6 Gy.

**Figure 4:**
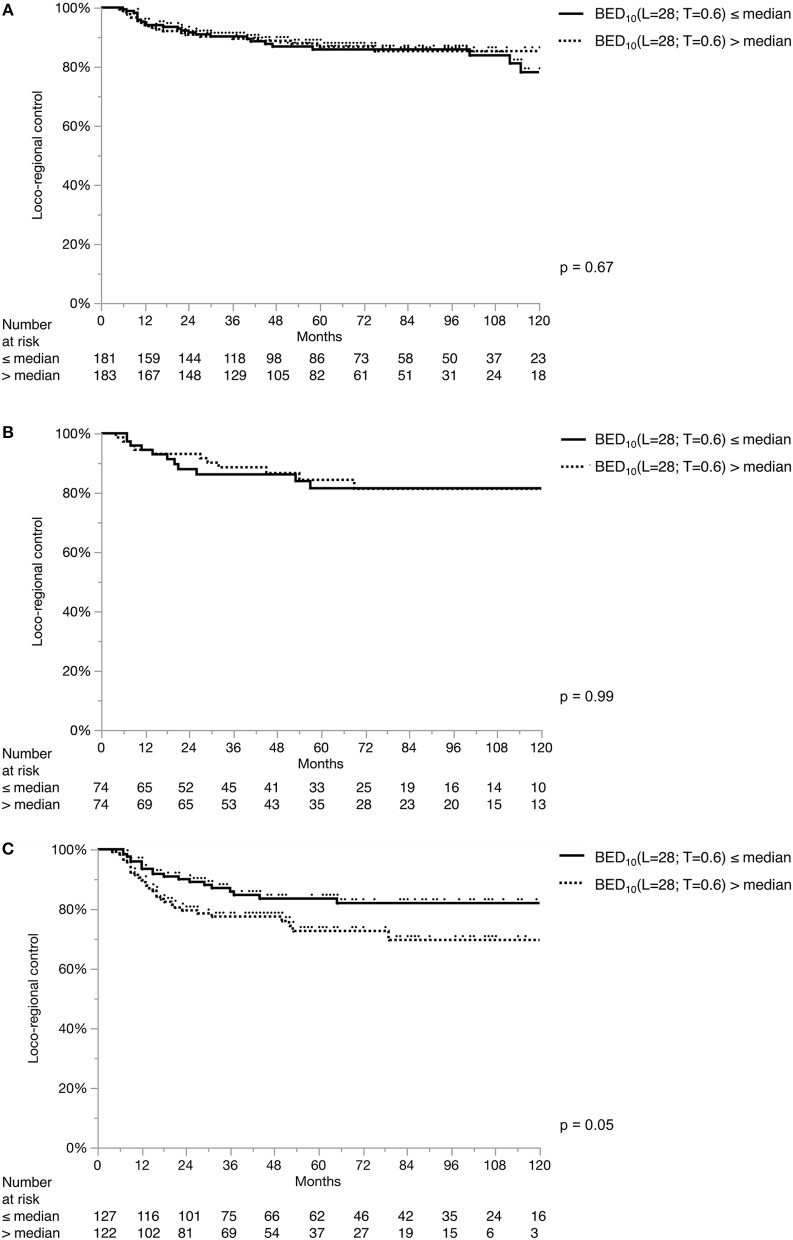
Loco-regional control separated by median BED_10_(L = 28; T = 0.6). Loco-regional control in T1a **(A)**, T1b **(B)**, and T2 **(C)** tumors. Each stage subgroup is dichotomized with its own median value: stage T1a: 70.6 Gy; stage T1b: 70.8 Gy; stage T2: 71.93 Gy.

Across all centers, the most frequently used fraction size was 2 Gy (66%). In a decreasing order; conventional fractionation (defined as > 1.8 and < 2.25 Gy) (69%), hypofractionation (25%), pure hyperfractionation (14) (5%) and partial hyperfractionation (e.g., concomitant boost) (1%) was utilized. Two centers used a single standard dose/fractionation schedule (2 and 2.75 Gy, respectively), whereas the remaining centers used various schedules (Supplementary Material 2). Concerning the composite impact of dose, fractionation and overall treatment time, Figures 3 and 4 demonstrate the LRC differences in each stage based on their median BED_10_(L = 2 5; T = 1) and BED_10_(L = 28; T = 0.6), respectively. There was no significant difference in LRC according to BED for either model except for higher BED_10_(L = 25; T = 1) yielding better LRC in T1a tumors (Figure 3A). Treatment technique, fraction size, total treatment time and BED were not normally-distributed in the whole cohort (Supplementary Material 1) and were heterogeneous among and within the institutions (Supplementary Material 2).

The results of the uni- and multivariate Cox proportional hazard models evaluating the potential prognostic value of various parameters for LRC are provided in Table 2. Male sex, T2 stage and ACI were associated with inferior LRC according to the univariate analyses. Male sex and the presence of ACI at the time of diagnosis were isolated as independent risk factors in multivariate analysis. Although not initially planned, based on these results, an exploratory analysis was performed to test the possible contribution of sex and ACI to OS. Neither male sex (HR: 1.62; 95% confidence interval [CI]: 0.93–3.19; *p* = 0.94) nor the presence of ACI (HR: 1.03; 95% CI: 0.76–1.41; *p* = 0.84) influenced OS. Similarly, no significant difference in median age in men and women was observed according to Wilcoxon test (both 65 years, *p* = 0.96).

**Table 2:** Uni- and multivariate Cox's proportional hazard models for loco-regional control.

	**Univariate analyses**		**Multivariate model**	
**Variable**	**HR (95% CI)**	***p***	**HR (95% CI)**	***p***
Age ≥ vs. <65 years	0.96 (0.67–1.38)	0.83	—	—
Male vs. female sex	3.49 (1.47–11.37)	<0.01	3.42 (1.44–11.17)	<0.01^*^
**Stage**				
T1b vs. T1a	1.19 (0.71–1.94)	0.49	1.00 (0.59–1.68)	0.99
T2 vs. T1a	1.62 (1.08–2.43)	0.02	1.34 (0.87–2.08)	0.18
T2 vs. T1b	1.36 (0.83–2.27)	0.22	1.34 (0.82–2.25)	0.25
ACI yes vs. no	1.66 (1.38–2.45)	<0.01	1.51 (1.01–2.28)	0.047^*^
IMRT vs. 2D-/3D-RT	0.84 (0.49–1.36)	0.49	–	–
BED_10_(L = 25; T = 1) > vs. ≤ 61.4	0.94 (0.65–1.35)	0.73	–	–
BED_10_(L = 28; T = 0.6) > vs. ≤ 70.8	1.37 (0.95–1.96)	0.09	1.23 (0.85–1.80)	0.27

2D-RT, two-dimensional radiotherapy; 3D-RT, three-dimensional conformal radiotherapy; ACI, anterior commissure involvement; BED, biologically equivalent dose; CI, confidence interval; HR, hazard ratio; IMRT, intensity-modulated radiotherapy; L, time lag; RT, radiotherapy; T, time factor;

* *Remaining p < 0.01 after backwards elimination*.

Out of 761 of patients, 3 underwent total laryngectomy due to organ dysfunction without any evidence of tumor persistence or recurrence (two patients initially treated with 2 Gy per fraction, the other one with 2.25 Gy per fraction). One hundred and nineteen patients (16%) experienced a biopsy-proven recurrence in a median time of 15 months (range: 4–148). Patterns of tumor recurrence and subsequent treatments are provided in Table 3. With a median follow-up of 32 months (range: 0–224) after tumor recurrence, OS at 2 and 5 years after recurrence were 67 and 60%, respectively. The 5-years OS after tumor recurrence were 68%, 76% and 43% in patients who originally had T1a, T1b, and T2 tumors, respectively (*p* < 0.02). The exact T and N stages at the time of recurrence were not obtained. Of the patients with tumor recurrence, 88% were treated with curative intent, all (*n* = 104) of them in the form of salvage surgery. Nine and 87 patients underwent partial (information about the partial laryngectomy types not available) and total laryngectomies, respectively. Adjuvant re-irradiation was required in 14.4% of these cases. The 2- and 5-years OS after salvage treatment were 75 and 67%, respectively. The 5-years OS after salvage treatment of patients with the initially T1a, T1b, and T2 tumors were 69, 84, and 56%, respectively (*p* = 0.17). Detailed information about the types of salvage surgery and related complications was not obtained. The 2- and 5-years larynx preservation rates (i.e., death with intact larynx censored) after initial diagnosis in T1a/1b/2 stages were 93/93/89% and 88/87/85%, respectively.

**Table 3:** Patterns of recurrence and treatments.

**Site of recurrence**	**Stage T1a**	**Stage T1b**	**Stage T2**
Total number of recurrences	47 (13%)^*^	23 (16%)	49 (20%)^*^
Local	40 (85%)	17 (74%)	35 (71%)
Isolated neck nodes	2 (4%)	3 (13%)	4 (8%)
Local + neck nodes	4 (9%)	3 (13%)	8 (16%)
Distant only	0	0	1 (2%)
Loco-regional + distant	1 (2%)	0	1 (2%)
**Treatment of recurrence**			
Treatment with curative intent	45 (96%)	21 (91%)	38 (78%)
Salvage S	38 (81%)	17 (74%)	34 (69%)
Salvage S + RT	7 (15%)	4 (17%)	4 (8%)
Salvage laryngectomy within S ± RT	43 (96%)	18 (86%)	35 (92%)
Total laryngectomy within laryngectomies (remaining cases underwent partial laryngectomy)	37 (86%)	18 (100%)	32 (91%)
Palliative chemotherapy	1 (2%)	1 (4%)	4 (8%)
Palliative RT	0	0	1 (2%)
Best supportive care	1 (2%)	1 (4%)	3 (6%)
Unknown/lost to follow-up	0	0	3 (6%)

RT, radiotherapy; S, surgery;

* *Percentage of the crude recurrence rate within the whole cohort*.

A total of 94 patients were diagnosed with metachronous SPCs. Of those; 28, 61 and 5 had head and neck SPCs, non-head and neck SPCs and both (synchronous), respectively. The incidence of SPC in 2, 5, and 10 years was 4, 10, and 21%, respectively. No association between fraction size and SPC risk was found: hazard ratio (per change in regressor over entire range): 1.48; 95% CI: 0.44–4.78; *p* = 0.52. Based on the results regarding the impact of sex on LRC and the lack of data about smoking and alcohol status, an exploratory analysis was performed to look for any difference in SPC among men and women, which may indirectly suggest a difference in the exposure to habitual carcinogens. However, no difference was observed (HR for SPC in men/women: 1.06; 95% CI: 0.56–2.27; *p* = 0.87).

## Discussion

The main objective of this pooled analysis was to report the outcome of stage I-II EGSCC patients treated at 10 university and teaching hospitals with definitive RT, in order to determine outcomes and identify possible prognostic factors. Our study confirms that RT of EGSCC results in favorable LRC and OS in line with the literature (4, 5). On multivariate analysis, independent negative prognostic factors for LRC were ACI and male sex, with the latter being a rather unexpected finding.

By contrast with other subsites of head and neck tumors where male sex has been consistently associated with poorer outcome, a survival advantage for male sex has been reported in larynx cancer (34, 35). It has been speculated that this might be due to differences in tumor localization with men being significantly more affected by glottic tumors while women presenting more often supraglottic cancers (36, 37). However, the presence of significant sex differences in our cohort solely composed of patients with glottic laryngeal cancer points to other possible factors, such as different history and behaviors of smoking and smoking cessation between men and women at the time of diagnosis, as well as during and after the treatment. On the other hand, as an indirect surrogate for exposure to carcinogens, no difference in the SPC incidence between men and women was observed. Still, we can not directly rule out the possibility of such a difference. It is also worth noting, that the OS and the distribution of age were not significantly different among men and women in our cohort.

In the EGSCC literature, the distribution of sex is nearly always descriptively reported, whereas its possible prognostic value is less frequently addressed. In two separate recent pooled analyses of National Cancer Database (USA) (28, 29), male sex was found to be a prognostic factor negatively influencing OS in the multivariate Cox proportional hazards models, whereas its impact for LRC was not investigated. In some studies male sex was associated (38–40) with poor LRC while this was not the case in others (10, 17, 18, 41–43), as based on univariate analyses. To the best of our knowledge, there are only other two retrospective studies, which reported on male sex as a poor prognostic factor for LRC emerging from the multivariate analysis (26, 27). In the age of biomarkers, sex as one of the most obvious phenotypic features can be the elephant in the room (44). In one way or another, it is important to further investigate this finding. The possible impact of genetic and hormonal factors on tumor control would expand our understanding of tumor biology and treatment response. On the other hand, in case of the lack of a direct causality between sex and oncologic outcome, but the identification of confounding factors such as behavioral differences (e.g., smoking and alcohol) among men and women, more emphasis would be given to modify these habits.

Some previous retrospective studies demonstrated that ACI is a poor prognostic factor for RT outcome (7–11), but these results are not consistent with the results of other studies (24). Some authors pointed out the possible underdosage of tumors with ACI close to the skin caused by the air-tissue interface (43). In our series, the finding of impaired LRC in the presence of ACI was reproduced. However, due to the lack of information about treatment volumes and details of treatment techniques, the underlying reason could not be identified. The impact of ACI should be further investigated, which may be integrated in the future staging algorithms and treatment algorithms.

Altered fractionation with shorter overall treatment time is known to be associated with better tumor control and survival benefit in head and neck cancer (45–50). In the last decade, three prospective randomized trials from Japan and Korea addressed fractionation specifically in EGSCC (17, 18, 25), favoring moderate hypofractionation (2.25 Gy), with disease outcome superiority and logistic benefits. On the other hand, contrary to what was expected, RTOG 9512 showed increased toxicity and futility with hyperfractionation in T2 glottic larynx cancer (19). Using accelerated RT, the results of the DAHANCA 6 trial showed a significant improvement in the loco-regional control of EGSCC with a hazard ratio of 0.60 (95% CI: 0.41–0.89) with a median follow-up of 14.5 years. There were no significant differences in long term toxicity between accelerated and normofractionated RT (16). Despite of the relatively large sample size, such an impact on oncologic outcome could not be reproduced in our cohort, probably due to the lack of normal distribution of fraction size, treatment time and BED. The only exception shown by the univariate log-rank test on the BED_10_(L = 25; T = 1) model yielding higher LRC in T1a tumors. Nevertheless, this may also be a result of multiple testing with two models in three tumor stages.

High SPC rates in head and neck cancer patients is a major problem. With each passing year, about 3% of the successfully treated patients are expected to develop a SPC (51, 52). In the SEER database analysis published by Rusthoven et al. (53), a reduced incidence SPC within the head and neck region was observed in patients treated with vs. without RT (hazard ratio: 0.71, 95% CI: 0.65–0.79; *p* < 0.01). The difference was still significant in laryngeal subsite on multivariate analysis. The authors suggested that RT had a preventative effect on transformation of the subclinical malignant foci. Our results about SPC incidence are consistent with the literature. Due to the lack of previously published data, we performed an exploratory analysis on fraction size and the incidence of SPC, and could not find any correlation at all. However, our median follow-up of 5 years is not long enough to observe any meaningful difference or exclude the long-term possibility of increased incidence of SPC. Moreover, the hypothesis regarding ablation of the premalignant foci with RT may be invalid or less prominent in the IMRT era, and if still present, this effect might be limited in the treatment of EGSCC, where elective nodal irradiation is often omitted. Data regarding the size of radiation portals, target volumes and whether elective nodal irradiation was performed was not collected in our pooled patient cohort.

Recent data suggests that IMRT may decrease the toxicity of RT for larynx cancer (54), although the approach of treating the whole larynx as a compartment is still widely used for EGSCC. It is based on the traditional conventional field design, which was established in an era where image guidance in RT was poor. Another reason was the laryngeal displacement due to swallowing movements during RT, which was later reported to be not a serious concern (55, 56). A combination of IMRT and modified target volumes (57) offers the potential to avoid the unnecessary dose to the healthy laryngeal tissue and especially to the carotid arteries (58, 59). With a newer technique developed by the Rotterdam group, it is even possible to apply 58.08 Gy in 16 fractions limited to the involved vocal cord with a significant dose reduction in the vicinity (60). The clinical results indicate no grade 3 toxicity, whereas the 2-year LRC and OS were 100% and 90%, respectively (61). When compared with a historical cohort, which was treated to the whole larynx (66/2 Gy), single vocal cord irradiation yielded less grade ≥2 acute toxicity (17 vs. 66%, *p* < 0.01) and lower voice-handicap index scores in all follow-up visits performed in regular short intervals until 18 months (*p* < 0.01). Based on these results, our group is about to start with a multi-center randomized phase III trial, which will compare voice quality after single vocal cord irradiation vs. transoral CO_2_ laser microsurgery in Tis and T1a N0 glottic cancer (NCT04057209).

The lack of a significant difference in OS between different initial T stages after salvage treatment can be explained by the patterns of recurrence. Most tumors recurred locally, which can be often successfully salvaged by total laryngectomy. On the other hand, the quality of life burden of such measures is well-known (62).

Our present study has limitations owing to its retrospective and multi-center nature, which predisposes the results to potential bias. Most importantly, we were not able to report on toxicity, smoking and alcohol consumption status due to the lack of consistent data. The wide range of dose-fractionations and techniques employed across 10 centers limits the ability of this type of analysis to identify a superior schedule, although our analysis did not detect differing outcomes based upon BED calculations. Last but not least, it should be noted that the heterogeneity of primary treatment preferences among participating centers presumably have an inevitable impact on the results. During the data acquisition in each center, the patients treated with RT were not systematically identified within the whole collective of patients (including those treated with primary surgery), who were diagnosed with EGSCC. Therefore, the quantification of the treatment patterns, which would indicate the institutional preferences, is not available.

## Conclusions

The results of our series demonstrate a negative impact of ACI on tumor control, indicating an additional prognostic value of ACI involvement beyond the current UICC TNM staging system for EGSCC. The less-expected and intriguing finding was the negative impact of male sex on tumor control. Further research is required in order to elucidate the true role of sex on oncological outcome in glottic laryngeal cancer or investigate any related behavioral factors, which may be modified for better oncologic outcome.

## Data Availability Statement

The anonymized datasets generated for this study are available on request to the corresponding author.

## Ethics Statement

The studies involving human participants were reviewed and approved by corresponding regional ethics committees of each institution. The patients/participants provided their written informed consent to participate in this study.

## Author Contributions

OE, RG, and MŞ: conception and design. OE, EE, CO, FC, GS, GH, LAd, LAn, MO, MS, MŞ, NK, OR, RP, and TS: collection of data. Drafting of the manuscript and approval of the final version by all co-authors.

### Conflict of Interest

The authors declare that the research was conducted in the absence of any commercial or financial relationships that could be construed as a potential conflict of interest.
