# Supplementary material for: Influencing Factors on Radiotherapy Outcome in Stage I-II Glottic Larynx Cancer—A Multicenter Study
Source: Front Oncol. 2019 Sep 20;9:932. doi: 10.3389/fonc.2019.00932 (PMC6763757; doi:10.3389/fonc.2019.00932)
Supplement: Supplementary file 1 [file Data_Sheet_1.ZIP › supplementary material 2.html]

Supplement 2
